# Cassava foliage affects the microbial diversity of Chinese indigenous geese caecum using 16S rRNA sequencing

**DOI:** 10.1038/srep45697

**Published:** 2017-04-06

**Authors:** Mao Li, Hanlin Zhou, Xiangyu Pan, Tieshan Xu, Zhenwen Zhang, Xuejuan Zi, Yu Jiang

**Affiliations:** 1 Tropical Crops Genetic Resources Institute, Chinese Academy of Tropical Agricultural Sciences, Danzhou 571737, Hainan, China; 2 College of Animal Science and Technology, Northwest A&F University, Yangling 712100, Shaanxi, China; 3 Institute of Tropical Agriculture and Forestry, Hainan University, Danzhou 571737, Hainan, China

## Abstract

Geese are extremely adept in utilizing plant-derived roughage within their diet. However, the intestinal microbiome of geese remains limited, especially the dietary effect on microbial diversity. Cassava foliage was widely used in animal feed, but little information is available for geese. In this study, the geese were fed with control diet (CK), experimental diet supplemented with 5% cassava foliage (CF5) or 10% (CF10) for 42 days, respectively. The cecal samples were collected after animals were killed. High-throughput sequencing technology was used to investigate the microbial diversity in the caecum of geese with different dietary supplements. Taxonomic analysis indicated that the predominant phyla were distinct with different dietary treatments. The phyla Firmicutes (51.4%), Bacteroidetes (29.55%) and Proteobacteria (7.90%) were dominant in the CK group, but Bacteroidetes (65.19% and 67.29%,) Firmicutes (18.01% and 17.39%), Proteobacteria (8.72% and 10.18%), Synergistete (2.51% and 1.76%) and Spirochaetes (2.60% and 1.46%) were dominant in CF5 and CF10 groups. The abundance of Firmicutes was negatively correlated with the supplementation of cassava foliage. However, the abundance of Bacteroidetes and Proteobacteria were positively correlated with the supplementation of cassava foliage. Our study also revealed that the microbial communities were significantly different at genus levels. Genes related to nutrient and energy metabolism, immunity and signal transduction pathways were primarily enriched by the microbiome.

Microbial community in gastrointestinal tract (GIT) related to the functions of host, including maintaining host health, improving performance, reducing environmental pollution, and ensuring food and animal products’ safety^1^,^2^,^3^,^4^,^5^. Meanwhile, fecal microbiome also reflects feed conversion, gut pathogen and parasite colonization and immune system activity of host^6^,^7^,^8^. Therefore, both gut and fecal microbiome play an important role for animals. However, feces are the final products of digestion, and fecal microbiome mainly affects the environment rather than host which composition is mainly determined by GIT original microbiome^9^. Furthermore, gut microbiome and host have interaction effect, and nutrition means could regulate gut microbiome in animal production. The small intestine, colon and caecum have similar roles in digestion and absorption of nutrient components. However, there are more microbes in caecum than other GIT2. The mechanism of caecum fiber digestion is similar with rumen, fiber fermentation by microbes eventually produces volatile fatty acids (mainly acetic acid, propionic acid and butyric acid) and ammonia, and then the intestinal epithelium absorbs these for host. Therefore, analyses of caecum microbiome represent a key area of nutrition research in poultry, and it would lead to an additional understanding of the microbial biodiversity and interactions with hosts.

Compared with duck or other family Anatidae poultry, geese have excellent ability of roughage utilization and adaptability. Even if comparable proventriculus, small intestine and caecum, geese’s GIT has a bigger and more powerful gizzard. The powerful gizzards can generate greatest forces for breaking down the roughage with high fiber content, such as forage, corn straw silage, wheat straw and rice byproducts^10^,^11^,^12^. Another pivotal reason is that geese have increasing effective caecum fermentation. The microbiome in caecum actively ferment and convert fiber contents into digestible components for animal hosts. The caecum is a complex ecosystem that harbors a wide variety of microbiome, which is an important factor for animal production. Therefore, it is necessary to study the gut microbial diversity in geese. However, only few studies have investigated the microbial diversity in the caecum of geese. Wang *et al*.^13^ reported that the dominant bacteria in the caecum of geese are related to *Pseudomonas* sp. and *Bifidobacterium* sp. by denaturing gradient gel electrophoresis (DGGE) fingerprints. Liu *et al*.^14^ analyzed the geese caecum microbial diversity using 16S rRNA clone library approach, which are dominantly occupied by *Clostridia*-related species (58.7%), followed by *Bacteroidetes* (26.9%) and *Erysipelotrichi* (11.2%). However, the DGGE technique fails to accurately represent the gut microbiome due to its low coverage, throughput and semi-quantitative features. In addition, the DGGE technique is time-consuming and insufficient to reflect the true diversity of a diverse gut microbiota. The 16S rRNA has been widely used to study and characterize bacterial community compositions because it can maximize the bacterial classification. However, the deficiencies of this technology are very obvious, and bacterial diversity is limited by depth of sequencing and cost. In recent years, high-throughput next-generation sequencing (HT-NGS) has become more sophisticated, and it provides large-scale analysis with unprecedented depths and coverages. Compared with conventional 16S rRNA technique, HT-NGS 16S rRNA technique can achieve high coverage, which can reflect the microbiome structure more accurately. HT-NGS technology has been used to study the intestinal microbiome of chickens, rabbits, goats and dairy cows^15^,^16^,^17^,^18^. However, to date, there are only few studies reported the microbial community in the caecum of geese by HT-NGS technology.

Cassava foliage is known as an agricultural by-product with high protein contents, gross energy and mineral elements, and it can be used as animal feed. It has been used as dietary in chickens and ducks with positive effects on the growth performance^19^. From the perspective of feed resource, little information is available regarding the utilization of cassava foliage for geese. Therefore, we aimed to investigate the effects of cassava foliage as a dietary supplement on microbial diversity in the caecum of geese using HT-NGS technology.

## Results

### Sequences

**Figure 1:**
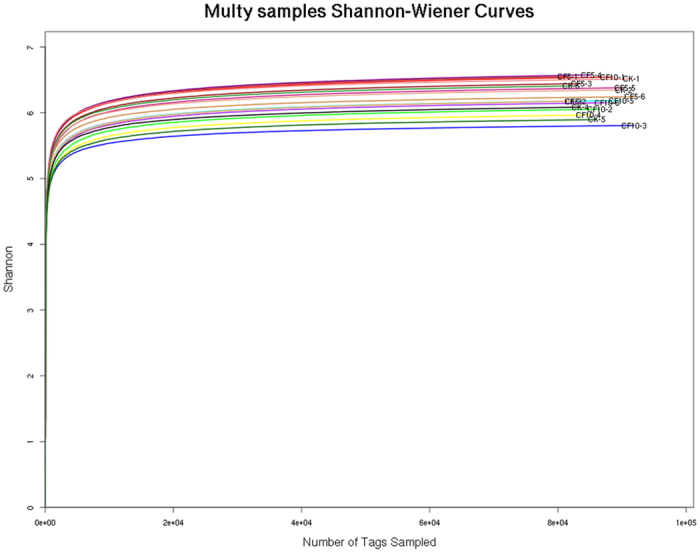
Shannon value and rarefaction curves of OUTs clustered at 97% sequence identity across different samples.

A total of 712,593,146 qualified sequences were obtained from the 18 samples from 18 geese caecum. These sequences included an average of 77,100 reads per caecum sample, and the length of unique tag N50 is 460 bp. (Table 2 and Table S1 and 2). Shannon value and Shannon rarefaction curves for each sample reached the saturation plateau (Table 2 and Fig. 1), indicating that the sampling effort had sufficient sequence coverage to accurately describe the bacterial composition of each group. Indices of bacterial richness based on OTUs were estimated by the method of Ace and Chao, and indices of bacterial diversity were determined using the method of Simpson and Shannon (Table 2). Among the 18 samples, a total of 200,365 OTUs were detected by our analysis, with an average of 11,131 OTUs per sample (Table 2).

### Taxonomic composition

**Figure 2:**
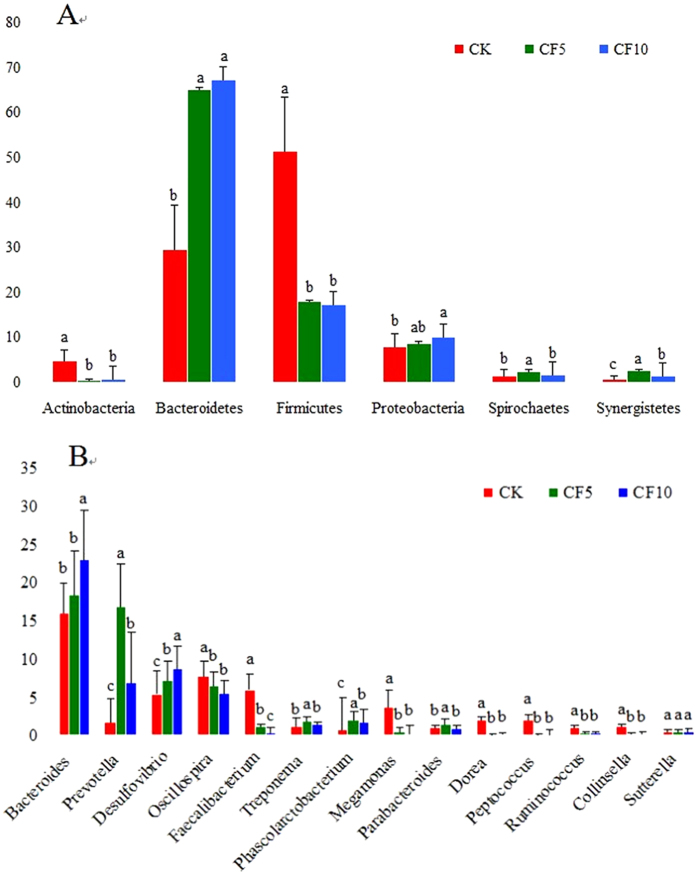
Effects of cassava foliage on the relative abundance (%reads) of (**A**) the most dominant phylum and (**B**) the most dominant genus in the cecal microbiome of geese. Error bars represent the SD of three samples. Boxes with a different letter above the error bars are significantly different at *P* < 0.01 by t-test analyses.CK represents control diet group; CF5 represents experimental diet which supplemented with 5% cassava foliage on the base of control diet; CF10 represents experimental diet which supplemented with 10% cassava foliage on the base of control diet. The same as below.

All sequences were classified from phylum to species based on the SILVA taxonomic database and using the analytical program QIIME. A total of 24 different phyla were detected from these samples. The Fig. 2 shows that the three groups had very dissimilar taxonomic compositions, even at the phylum-level distribution. Firmicutes (51.4%), Bacteroidetes (29.55%) and Proteobacteria (7.90%) were the dominant phyla in the CK (control diet) group. Bacteroidetes, Firmicutes, Proteobacteria, Synergistete and Spirochaetes were the dominant phyla in the CF5 (experimental diet was supplemented with 5% cassava foliage on the base of control diet) group, representing 65.19%, 18.01%, 8.72%, 2.51% and 2.60% of the total reads, respectively. Bacteroidetes, Firmicutes, Proteobacteria, Synergistete and Spirochaetes were the most commonly detected phyla in the CF10 (experimental diet was supplemented with 10% cassava foliage on the base of control diet) group, accounting for 67.29%, 17.39%, 10.18%, 1.76% and 1.46% of the total reads, respectively.

**Figure 3:**
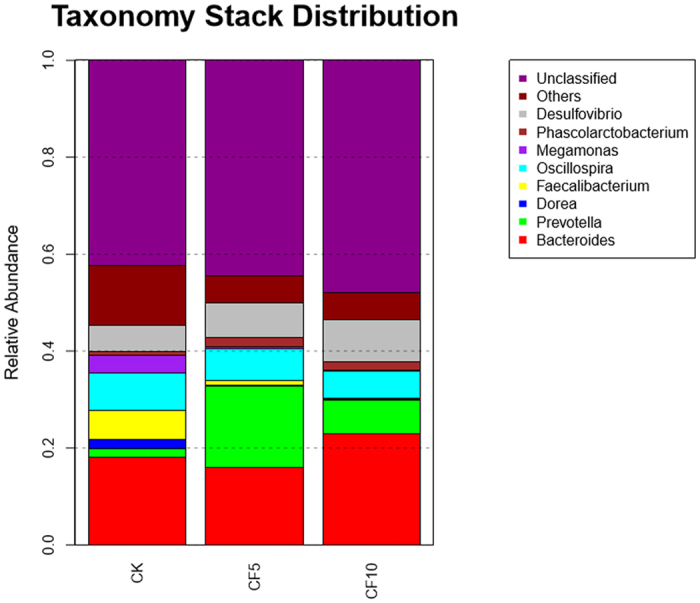
Genus-level composition of the cecal microbiome of geese. A color-coded bar plot shows the average bacterial genus distribution in different treatment groups.

**Figure 4:**
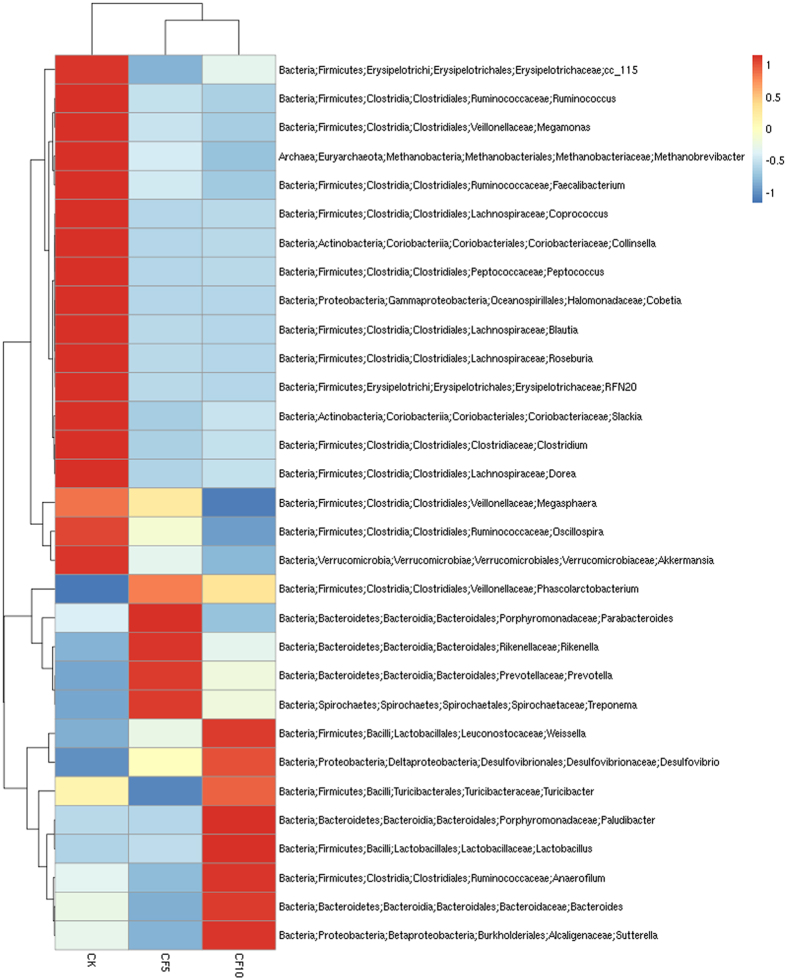
A heat map of the microbial composition in the caecum of geese at the genus level. The heat map indicates the relative abundance of each genus in different treatment groups.

At the genus level, the detected sequences could be assigned into 163 different genera. The most abundant genera (the relative abundance of more than 1% of the three libraries) among the libraries suggested the importance of bacteria (Figs 2, 3 and 4). In the CK group, *Bacteroides, Oscillospira, Faecalibacterium, Desulfovibrio, Megamonas, Dorea, Peptococcus, Prevotella, Treponema, Collinsella, Blautia, Parabacteroides* and *Ruminococcus* were the dominant genera, representing 16%, 7.73%, 6.02%, 5.43%, 3.66%, 2.04%, 1.96%, 1.71%, 1.14%, 1.09%, 1.07%, 1 and 1% of the total sequences, respectively. In the CF5 group, *Bacteroides, Prevotella, Desulfovibrio, Oscillospira, Phascolarctobacterium, Treponema, Parabacteroides* and *Faecalibacterium* were the dominant genera, representing 18.05%, 16.86%, 7.11%, 6.42%, 2.01%, 1.83%, 1.43% and 1.09% of the total sequences, respectively. In the CF10 group, the most abundant sequences were related to *Bacteroides, Desulfovibrio, Prevotella, Oscillospira, Phascolarctobacterium* and *Treponema,* representing 22.94% 8.72% 6.89% 5.56% 1.65% and 1.38% of the sequences, respectively. We also noticed that there were many unclassified and uncultured bacteria in the samples from the CK, CF5 and CF10 groups, representing 57.63%, 55.50% and 52.03% of the total sequences, respectively.

**Figure 5:**
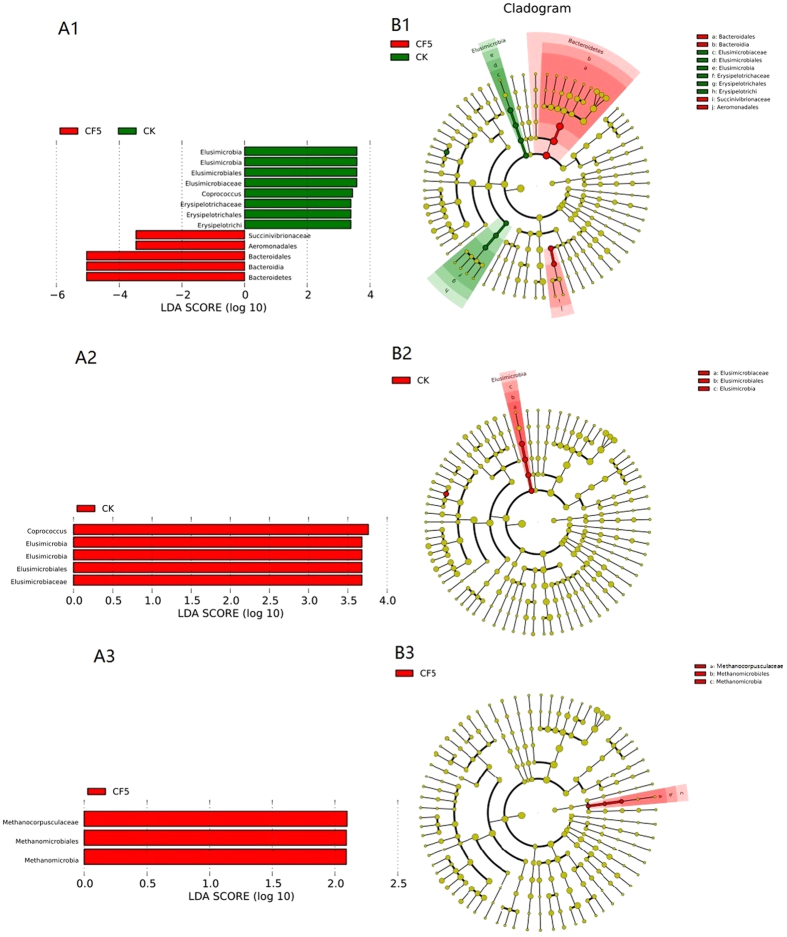
LEfSe identified the most differentially abundant taxons between CK and CF5 (**A1**,**B1**), CK and CF10 (**A2**,**B2**), CF5 and CF10 (**A3**,**B3**). Taxonomic cladogram obtained from LEfSe analysis of 16 S sequences (relative abundance ≥0.5%). (Red) CF5-enriched taxa, (Green) taxa enriched in CK (**B1**); (Red) CK-enriched taxa (**B2**); (Red) CF5-enriched taxa (**B3**). The brightness of each dot is proportional to its effect size. CK-enriched taxa are indicated with a positive LDA score (green), and taxa enriched in CF5 have a negative score (red) (**A1**); taxa enriched in CK have a negative score (red) (**A2**); taxa enriched in CF5 have a negative score (red) (**A3**). Only taxa meeting an LDA significant threshold >2 are shown.

To identify the specific bacterial taxa associated with cassava foliage, we compared the caecum microbiota in CK and CF5, CK and CF10, and CF5 and CF10 using LEfSe. Figure 5 shows a representative cladogram of the structure of the caecum microbiota and their predominant bacteria, showing the greatest differences in taxa between the three groups. The data indicated that two Order (*Bacteroidales* and *Aeromonadales*) and three Family (Elusimicrobiaceae, Erysipelotrichaceae and Succinivibrionaceae) belonged to the four dominant phyla (Bacteroidetes, Elusimicrobia, Firmicutes and Proteobacteria) in CK and CF5 (Fig. 5A1). One genus (*Coprococcus*) and one Family (Elusimicrobiaceae) belonged to the two dominant phyla (Elusimicrobia and Firmicutes) in CK and CF10 groups (Fig. 5A2). One Family (Methanocorpusculaceae) belonged to dominant phyla Euryarchaeota of Archaea in CF5 and CF10 groups (Fig. 5 A3). These different taxa could be used as distinguishing biomarkers. The changes in the caecum microbiota treated with cassava foliage were also explored using the Mann-Whitney U test at different taxon levels. At the phylum level, Bacteroidetes, Firmicutes, Proteobacteria, Synergistete and Spirochaetes were the most predominant phyla in the cassava foliage treated samples.

### The effect of cassava foliage on the relative abundance and diversity of microbial communities

Figure 2 reveals a profound change in microbial composition at the phylum and genus levels was induced by different treatments. The treatments of cassava foliage showed a statistically significant effect on the relative abundance of Bacteroidetes (*P* < 0.05), which was the most abundant phylum in cecal microbiome and increased with the addition of cassava foliage. In contrast, the relative abundance of Firmicutes, the second abundant phylum, was significantly decreased with the addition of cassava foliage (*P* < 0.05). The same trend was observed in Actinobacteria, the abundance of which was significantly decreased with the addition of cassava foliage (*P* < 0.05). The relative abundances of Proteobacteria, Synergistetes and Spirochaetes were increased with the addition of cassava foliage. The relative abundance of Proteobacteria in the CF10 group was significantly higher than that of the CK group (*P* < 0.05), and the relative abundances of Synergistetes and Spirochaetes in the CF5 group were significantly higher compared with the CK group (*P* < 0.05).

**Figure 6:**
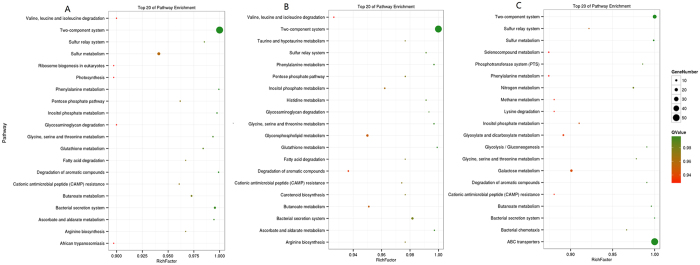
KEGG enrichment analysis of the difference groups. (**A**): CK-VS-CF5; (**B**): CK-VS-CF10; (**C**): CF5-VS-CF10. Rich Factor: The ratio of the number of differentially expressed genes and the total number genes which located in the pathway genes. The greater the Factor Rich, the higher the degree of enrichment. P-value closer to 0, the more significant enrichment.

In the present study, we found that the relative abundances of genera were also affected by the addition of cassava foliage. Figure 2 shows the 14 most abundant genera. The relative abundances of *Bacteroides, Prevotella, Desulfovibrio, Treponema* and *Phascolarctobacterium* were significantly increased with the addition of cassava foliage. However, the relative abundances of *Oscillospira, Faecalibacterium, Megamonas, Dorea, Peptococcus, Collinsella* and *Ruminococcus* were significantly decreased (*P* < 0.05). The relative abundance of *Parabacteroides* in the CF5 group was significantly higher compared with the CK and CF10 groups (*P* < 0.05). However, the relative abundance of *Sutterella* was not significantly different among all groups. Based on the functionality prediction, we detected a clear difference in the KEGG Orthologs (KO) composition in caecum. There were 104,109 and 87 differential enrichment KEGG pathways between CK and CF5, CK and CF10, CF5 and CF10, respectively. Figure 6 shows the top 20 pathway enrichment of different groups. Cecal microbial pathways were also detected, which were related to amino acid metabolism pathways (e.g. valine, leucine and isoleucine degradation, phenylalanine metabolism, glycine, serine and threonine metabolism, glutathione metabolism, arginine biosynthesis, taurine and hypotaurine metabolism, histidine metabolism, glutathione metabolism, nitrogen metabolism, lysine degradation), sugar and nucleotide sugar metabolism pathways (e.g. pentose phosphate pathway, glycosaminoglycan degradation, ribosome biogenesis in eukaryotes, phosphotransferase system (PTS), glycolysis/gluconeogenesis, galactose metabolism), fatty acid degradation pathways (e.g. fatty acid degradation, butanoate metabolism, degradation of aromatic compounds, glyoxylate and dicarboxylate metabolism), immunity and signal transduction (e.g. cationic antimicrobial peptide (CAMP), ascorbate and aldarate metabolism, African trypanosomiasis, two-component system, inositol phosphate metabolism, carotenoid biosynthesis, seleno compound metabolism), bacterial proliferation and colonization (e.g. bacterial secretion system and bacterial chemotaxis), sulfur metabolism pathways (e.g. sulfur relay system and sulfur metabolism), energy metabolism (methane metabolism), respectively. Notably, the two-component system pathway showed significant difference in the three groups, and the ABC transporter pathway showed significant difference in CF5 and CF10 groups.

## Discussion

Many studies have used high-throughput sequencing to investigate the gut microbial diversity of poultry. These studies on microbiome have shown that Firmicutes, Bacteroidetes and Proteobacteria are numerically the most dominant phyla in the cecal microbiome of chickens^20^,^21^,^22^,^23^. In this study, for the first time, we identified the microbial diversity of cecal samples from geese using high-throughput sequencing, and then the effect of cassava foliage on such diversity was studied. At the phylum level, Bacteroidetes, Firmicutes, Proteobacteria, Synergistete, Spirochaetes and Actinobacteria were identified as the dominant bacteria in the cecal microbiome of geese. However, Liu *et al*.^14^ analyzed the microbial diversity of geese caecum at the class level using 16S rRNA clone library approach, and the cecal microbiome of geese are dominantly occupied by 58.7% *Clostridia* (Firmicutes), 26.9% *Bacteroidetes* (Bacteroidetes) and 11.2% *Erysipelotrichi* (Firmicutes), showing similar results with the cecal microbiome of chickens. Despite the limited number of analyzed sequences, the results provided a valuable insight into a poorly understood microbial ecosystem of goose caecum. Interestingly, Bacteroidetes is found as the most abundant bacteria in the duck caecum, and the dominant phyla high to low are Bacteroidetes, Firmicutes and Proteobacteria^24^. Similar result has been reported in caecum 16 S rRNA clone library approach of turkeys, and the dominant phyla high to low are Bacteroidetes (54%), Firmicutes (30%), Proteobacteria (3%) and Deferribacteres (3%)^25^. The microbial diversity of goose caecum was similar with that of ducks and turkeys, but it was different from that of chickens. From the perspective of evolution, geese and ducks are both poultry of Anseriformes, and they have closer relationship than chickens and turkeys. However, from the perspective of feeding, the geese and turkeys have stronger ability of roughage utilization compared with chickens and ducks. Furthermore, geese and ducks live in water. Therefore, the microbial diversity of gut could be affected by all genetic, dietary and environmental conditions.

In the present study, there were evident differences in the microbial composition among different treatments of cassava foliage. The phylum Bacteroidetes was the most abundant bacteria and its abundance was increased with the addition of cassava foliage. The abundance of phylum Bacteroidetes in the CF5 and CF10 groups was significantly higher than that in the CK group, whereas the abundance of Firmicutes in the CF5 and CF10 groups was significantly lower than that in the CK group. The cassava foliage diet contained higher fiber content than the control diet, leading to increased abundance of Bacteroides and decreased abundance of Firmicutes. This result was consistent with the study on intestinal microbiome in ducks, showing increased abundance of Bacteroides and decreased abundance of Firmicutes in the caecum^24^. The similar dietary effects on intestinal microbiome with a higher abundance of Bacteroidetes and a lower abundance of Firmicutes have been reported in rabbits, goats and humans, and the different nutrient compositions might lead to the different biodiversities^16^,^17^,^26^. In the present study, the abundance of Proteobacteria in the CF5 and CF10 groups was significantly higher than that in the CK group. This finding was consistent with the studies on ducks and goats^17^,^24^, but inconsistent with the studies on chickens. Such a discrepancy could be caused by species-specific differences. The phylum Synergistetes, which is known for its ability to degrade amino acids and pyruvate, was one of the most abundant bacteria in our study. To date, no study has reported Synergistetes as the dominant bacteria in the gut microbiome of chickens or ducks. Our finding indicated that Synergistetes was unique to geese and could play a key role in cecal digestion. However, further studies are still necessary to thoroughly understand the effects of cassava foliage on the abundance of these bacteria.

In our study, we investigated the effect of cassava foliage on the population structure at the genus level. Among the different genera, *Bacteroides* was the most abundant bacteria in goose caecum. Previous studies have shown that the microbial diversity in chicken caecum was mainly dominated by Bacteroides, which was consistent with our study^12^,^23^. *Bacteroides* is thought to play a fundamental role in the breakdown of complex polysaccharides, starch and cellulose into simpler compounds^27^. The treatments of cassava foliage increased the abundance of *Bacteroides. Prevotella* was another abundant genus in our study. Previous studies have shown that *Prevotella* is the most abundant bacterial genus in the rumens of goats and bovine, both of which have strong ability of roughage utilization^17^,^28^. Here, we found that the abundance of *Prevotella* tended to increase with the addition of cassava foliage, which was consistent with previous studies. Additionally, this observation was in agreement with a recent study by Jami *et al*.^29^, which shows that the genus *Prevotella* becomes the dominant bacteria in the bovine rumen with high-fiber diets. *Prevotella* has important role in the utilization of carbohydrates within the gut microbial ecosystem, and its fermented products are mainly acetic acid, succinic acid, isobutyric acid, isovaleric acid and lactic acid, which can be used by the animal hosts. This might explain why the abundance of *Prevotella* was changed by treatments of cassava foliage.

Notably, we detected a large number of microbiome in goose caecum, which belonged to unclassified and uncultured genera based on the current 16 S RNA gene sequence database. This finding suggested that geese might possess specific intestinal microbiome, reflecting the fact that few studies of this type of poultry have been previously conducted. Additionally, the relative abundances of unclassified and uncultured bacteria were decreased with the addition of cassava foliage, indicating that the microbiome of geese with more roughage could be better classified compared with the geese with less roughage. Further studies are required to better characterize these unknown bacteria and their special functions in the hosts.

The microbial diversity of geese gut may present many important functions, which are essential to geese life. In this study, we made prediction based on KO. The results indicated that the most abundant functional categories were associated with amino acid metabolism, sugar and nucleotide sugar metabolism, fatty acid degradation, energy metabolism, immunity and signal transduction, bacterial proliferation and colonization. Utilization of amino, sugar and nucleotide sugar is important for geese metabolism and growth. The amino acid metabolism pathway is specifically responsible for breaking down protein to amino acids or peptides. The sugar metabolism pathway and fatty acid degradation pathway are specifically responsible for digesting cellulose or other dietary fiber to volatile fatty acids and absorbing. The nucleotide sugar metabolism is crucial for purine and pyrimidine generation, which is vital substrate for DNA or RNA synthesis. These biochemical processes might be related to the high-energy requirements and high metabolism rate, which are responsible for energy metabolism pathway. The pathways of bacterial proliferation and colonization play crucial roles in gut colonization and host adhesion, infection and biosynthesis of fimbriae, flagella, outer membrane, metabolic and lipopolysaccharides. In the present study, the two-component system pathway showed significant difference in the three groups, which was one of immunity and signal transduction pathways. The two-component system with sensor kinase and response regulator modulates gene expression based on environmental stimulus (e.g. temperature, pH, osmotic level, toxicity and nutrients)^2^,^30^. Cassava foliage treatments up-regulated the expressions of genes responsible for two-component system pathway compared with control, and the differentiation may be caused by composition and amount of carbohydrate. In addition, the ABC transporter pathway showed significant difference in two cassava foliage treatments. Major functions of ABC transporters include the transport of lipids, bile salts, toxic compounds, and peptides, which maintain host health and boost the immune^31^. However, the effects of cassava foliage on the cecal microbial and functions remain unclear, and the actual actions may wait for more efforts.

Taken together, our study, based on 16 S rRNA gene sequencing, reported the overall composition of the microbial ecosystem in the caecum of geese with different diets. Our data revealed that cassava foliage treatment had significant effects on the microbial community in the caecum of geese. Genes related to nutrient and energy metabolism, immunity and signal transduction pathways were significantly expressed by the microbiome. These observations provided a better understanding of how the microbial ecology in the caecum of geese was affected by diet.

## Materials and Methods

### Experimental design and sampling

All protocols of animal handling and sampling were approved by the Animal Care and Use Committee of Chinese Academy of Tropical Agricultural Sciences (CATAS), and all efforts were made to minimize the suffering of animals according to recommendations proposed by the European Commission (1997). The study was carried out in accordance with the approved protocol. All methods were conducted in accordance with relevant guidelines. A total of 108 male Hainan indigenous geese (28 days old) with similar body weight were randomly divided into three groups with six cages of six geese per group. The geese were fed for 42 days with different diets in this trail, and Table 1 lists the dietary compositions. CK group was fed with control diet, and CF5 or CF10 group was fed with experimental diet supplemented with 5% cassava foliage (CF5) or 10% cassava foliage (CF10), respectively (Table 1). For the consistency of energy and protein levels, we adjusted several components, such as fish meal. After 12-h starvation at age of 70 days old, all the birds were individually weighed, and one bird per cage (with body weight closest to the mean cage weight) was selected and sacrificed. Birds were euthanized by cervical dislocation. Samples were aseptically scrapped from caecum and placed into a sterile glass slides. All samples were immediately stored at −80 °C until further analysis[Table t2].

### DNA extraction and 16S rRNA gene sequencing

Total genomic DNA was extracted from cecal samples using a Stool DNA Kit (OMEGA Bio-Tek, Norcross, GA, USA) according to the manufacturer’s instructions. V3-V4 regions of bacterial 16S rRNA gene (from 341 to 806) were amplified from extracted DNA using barcoded primers 349 F (5′- CCTACGGGNBGCASCAG -3′) and 806 R (5′-GACTACNVGGGTATCTAATCC-3′). PCR was performed in a 20-μL reaction system containing 0.8 μL of each primer, 10 ng template DNA, 4 μL 5 × FastPfu buffer, 2 μL 2.5 mM dNTPs and 0.4 μL FastPfu polymerase, and experiments were conducted in triplicate. Briefly, following an initial denaturation step at 95 °C for 5 min, the amplifications were carried out with 27 cycles at a melting temperature of 95 °C for 30 sec, an annealing temperature of 50 °C for 30 sec, and an extension temperature of 72 °C for 45 sec. Finally, an extra extension step at 72 °C for 10 min was performed. The amplicons were pooled, purified and then quantified using Nanodrop (Thermo Scientific, USA). Subsequently, next-generation sequencing was performed by Illumina Hiseq 2500 PE250, which was conducted by Genedenovo Inc. (Guangzhou, China).

### Quality control

Reads filtering: (1) removing reads containing more than 10% of unknown nucleotides (N); (2) removing reads containing less than 80% of bases with quality (Q-value) > 20.

Tag assembling and abundance statistics: The filtered reads were then assembled into tags according to overlap between paired-end reads with more than 10-bp overlap and less than 2% mismatch. The software MOTHUR^32^ was used to remove the redundant tags to get unique tags. The obtained unique tags were then used to calculate the abundance.

### Bioinformatics and statistical analysis

The high-quality sequences were clustered into operational taxonomic units (OTUs) defined at 97% similarity. These OTUs were applied for diversity (Shannon and Simpson), richness (Ace and Chao) and rarefaction curve analyses using MOTHUR^32^. Taxonomic assignments of OTUs that reached the 97% similarity level were made using (quantitative insights into microbial ecology) QIIME software package through comparison with the SILVA^33^, Greengene^34^ and RDP^35^ databases. A heat map was generated using the heat map function of the R (http://www.r-project.org/) and genus information for the three groups. Linear discriminant analysis (LDA) effect size (LEfSe) method was used to identify the most differentially abundant taxons between groups, which would help discover biomarkers^36^. The predicted KOs were summarized to functional categories at the genus level. Groups were compared using the Statistical Analysis of Metagenomic Profile package STAMP (http://kiwi.cs.dal.ca/Software/STAMP)^37^. Statistical analysis was performed using t-tests with the SPSS software (version 19.0 for Windows, SPSS Inc., Chicago, IL, USA). *P* < 0.05 was considered statistically significant.

### Nucleotide sequence accession numbers

Sequences of this project have been deposited into the NCBI nucleotide database under accession number SRA544802.

## Additional Information

**How to cite this article:** Li, M. *et al*. Cassava foliage affects the microbial diversity of Chinese indigenous geese caecum using 16S rRNA sequencing. *Sci. Rep.* **7**, 45697; doi: 10.1038/srep45697 (2017).

**Publisher's note:** Springer Nature remains neutral with regard to jurisdictional claims in published maps and institutional affiliations.

## Supplementary Material

Supplementary Tables

## Figures and Tables

**Table 1:** Ingredient and nutrient composition (%, as feed) of the experimental diets.

Items	CK	CF5	CF10
Ingredient (%)			
Corn	62	58.5	50.3
Soybean meal	22	21	20
Wheat bran	9	7.5	7
Cassava foliage		5	10
Vegetable Oil	0.5	1.5	4
Fish meal			3
Limestone powder	2	2	1.5
Calcium hydrogen phosphate	0.2	0.2	
DL-Met	0.3	0.3	0.2
Premix compound	4	4	4
Total	100	100	100
Nutrient composition			
ME (MJ/kg)	11.27	11.3	11.34
CP (%)	16.48	16.53	16.47
CF (%)	3.04	5.01	6.93
Lys (%)	0.8	0.8	0.8
Met (%)	0.45	0.45	0.45
Ca (%)	0.8	0.8	0.8
P (%)	0.5	0.5	0.5

ME: Metabolizable energy, CP: Crude protein, CF: Crude fiber, Lys: lysine, Met: Methionine, Ca: Calcium, P: Phosphorus.

**Table 2 t2:** Diversity estimation of the 16S rRNA gene libraries of the goose gut from the sequencing analysis^a^.

Sample ID^b^	Reads	OTU	Chao	Ace	Shannon	Simpson
CK-1	83,124	11,804	33,282	59,669	6.51	0.0076
CK-2	79,931	11,567	33,033	60,570	6.35	0.0122
CK-3	75,341	10,869	31,631	60,520	6.18	0.0187
CK-4	70,146	10,116	29,761	56,110	6.08	0.0160
CK-5	73,894	9,730	25,895	47,235	5.90	0.0188
CK-6	73,334	11,210	31,269	56,040	6.41	0.0102
CF5–1	75,345	12,296	33,400	58,588	6.55	0.0095
CF5–2	71174	10524	30621	54,201	6.17	0.0129
CF5–3	73,430	11,785	33,281	59,620	6.44	0.0114
CF5–4	77291	13,036	36,996	66,589	6.58	0.0098
CF5–5	79482	12116	33277	60234	6.38	0.0102
CF5–6	84,883	10,372	26,924	44890	6.24	0.0101
CF10–1	81,096	12,829	36,135	65,404	6.54	0.0095
CF10–2	76,117	10,685	28952	52,824	6.05	0.0164
CF10–3	80,438	8,885	22,792	39,794	5.81	0.0169
CF10–4	74,099	10,327	29,455	53,691	5.97	0.0204
CF10–5	80,233	11,205	30,620	52,934	6.19	0.0135
CF10–6	78,459	11,009	29,683	52,174	6.15	0.0156

^a^OTUs were defined at 3% dissimilarity. The richness estimators (ACE and Chao) and diversity indices (Shannon and Simpson) were calculated.

^b^Samples in the CK group included CK1, CK2, CK3, CK4, CK5 and CK6; samples in the CF5 group included CF5–1, CF5–2, CF5–3, CF5–4, CF5–5 and CF5–6; samples in the CF10 group included CF10–1, CF10–2, CF10–3, CF10–4, CF10–5 and CF10–6.
