# Corrigendum: Cassava foliage affects the microbial diversity of Chinese indigenous geese caecum using 16S rRNA sequencing

**DOI:** 10.1038/srep46837

**Published:** 2017-06-15

**Authors:** Mao Li, Hanlin Zhou, Xiangyu Pan, Tieshan Xu, Zhenwen Zhang, Xuejuan Zi, Yu Jiang

Scientific Reports 7: Article number: 45697; 10.1038/srep45697 published online: 04 06 2017; updated: 07 15 2017.

The Acknowledgements section in this Article is incomplete.

“This study was funded by the Central Public-interest Scientific Institution Basal Research Fund for Chinese Academy of Tropical Agricultural Sciences (No.17CXTD-18, 1630032016020), the Special Fund for Agro-Scientific Research in the Public Interest, China (grant no. 201303143) and the China Agriculture Research System (grant no. CARS-12)”.

should read:

“This study was funded by the Central Public-interest Scientific Institution Basal Research Fund for Chinese Academy of Tropical Agricultural Sciences (No.17CXTD-18,1630032017078,1630032016020), the Special Fund for Agro-Scientific Research in the Public Interest, China (grant no. 201303143) and the China Agriculture Research System (grant no. CARS-12)”.

