# Supplementary material for: Cassava foliage affects the microbial diversity of Chinese indigenous geese caecum using 16S rRNA sequencing
Source: Sci Rep. 2017 Apr 6;7:45697. doi: 10.1038/srep45697 (PMC5382919; doi:10.1038/srep45697)
Supplement: Supplementary Tables [file srep45697-s1.doc]

**Additional files**

**Table S1.** Information of Tags

| Sample ID | Assemble | | | | | |
| --- | --- | --- | --- | --- | --- | --- |
|  | Number * | Total length(bp) | Max length | Min length | N50(bp) | N90 |
| CK-1 | 91474 | 41210633 | 479 | 301 | 446 | 440 |
| CK-2 | 90219 | 41106670 | 489 | 301 | 460 | 440 |
| CK-3 | 82336 | 37595298 | 483 | 301 | 460 | 442 |
| CK-4 | 83527 | 38198961 | 480 | 306 | 460 | 440 |
| CK-5 | 86074 | 39266719 | 480 | 304 | 460 | 440 |
| CK-6 | 82079 | 37373731 | 482 | 304 | 460 | 441 |
| CF5-1 | 81584 | 37267840 | 483 | 304 | 460 | 442 |
| CF5-2 | 82825 | 37910935 | 474 | 301 | 460 | 443 |
| CF5-3 | 83641 | 38207771 | 486 | 304 | 460 | 442 |
| CF5-4 | 85219 | 38987128 | 479 | 304 | 460 | 442 |
| CF5-5 | 90483 | 41436061 | 477 | 310 | 460 | 442 |
| CF5-6 | 91988 | 42024402 | 468 | 305 | 460 | 442 |
| CF10-1 | 88546 | 40515099 | 474 | 304 | 460 | 442 |
| CF10-2 | 86520 | 39506619 | 486 | 301 | 460 | 440 |
| CF10-3 | 91840 | 42028620 | 489 | 303 | 460 | 442 |
| CF10-4 | 84804 | 38643400 | 475 | 306 | 460 | 440 |
| CF10-5 | 89873 | 41173673 | 489 | 301 | 460 | 442 |
| CF10-6 | 87636 | 40139586 | 482 | 303 | 460 | 441 |

*Number： Number of Tags after filtering and trimming

**Table S2**. Information of Unique Tags

| Sample ID | unique tags | | | | | |
| --- | --- | --- | --- | --- | --- | --- |
|  | Number * | Total length(bp) | Max length | Min length | N50(bp) | N90(bp) |
| CK-1 | 83124 | 37427632 | 479 | 301 | 443 | 440 |
| CK-2 | 79931 | 36385522 | 489 | 301 | 460 | 440 |
| CK-3 | 75341 | 34379130 | 483 | 301 | 460 | 442 |
| CK-4 | 70146 | 32035035 | 480 | 306 | 460 | 440 |
| CK-5 | 73894 | 33682587 | 480 | 304 | 460 | 440 |
| CK-6 | 73334 | 33365863 | 482 | 304 | 460 | 441 |
| CF5-1 | 75345 | 34406955 | 483 | 304 | 460 | 442 |
| CF5-2 | 71174 | 32553086 | 474 | 301 | 460 | 442 |
| CF5-3 | 73430 | 33522089 | 486 | 304 | 460 | 442 |
| CF5-4 | 77291 | 35339245 | 479 | 304 | 460 | 442 |
| CF5-5 | 79482 | 36377795 | 477 | 310 | 460 | 442 |
| CF5-6 | 84883 | 38760534 | 468 | 305 | 460 | 441 |
| CF10-1 | 81096 | 37089105 | 474 | 304 | 460 | 442 |
| CF10-2 | 76117 | 34736615 | 486 | 301 | 460 | 440 |
| CF10-3 | 80438 | 36787106 | 489 | 303 | 460 | 442 |
| CF10-4 | 74099 | 33746467 | 475 | 306 | 460 | 440 |
| CF10-5 | 80233 | 36740312 | 489 | 301 | 460 | 442 |
| CF10-6 | 78459 | 35912228 | 482 | 303 | 460 | 441 |

*Number: Number of unique Tags after removing the redundant sequences
